# An Innovative Random-Forest-Based Model to Assess the Health Impacts of Regular Commuting Using Non-Invasive Wearable Sensors

**DOI:** 10.3390/s23063274

**Published:** 2023-03-20

**Authors:** Mhd Saeed Sharif, Madhav Raj Theeng Tamang, Cynthia H. Y. Fu, Aaron Baker, Ahmed Ibrahim Alzahrani, Nasser Alalwan

**Affiliations:** 1 Intelligent Technologies Research Group, ACE, UEL, University Way, London E16 2RD, UK; 2 School of Psychology, UEL, Water Lane, London E15 4LZ, UK; 3 Computer Science Department, Community College, King Saud University, Riyadh 11437, Saudi Arabia

**Keywords:** EEG sensors, stress assessment, blood pressure, commuting, wearable sensors, predictive models

## Abstract

Regular commutes to work can cause chronic stress, which in turn can cause a physical and emotional reaction. The recognition of mental stress in its earliest stages is very necessary for effective clinical treatment. This study investigated the impact of commuting on human health based on qualitative and quantitative measures. The quantitative measures included electroencephalography (EEG) and blood pressure (BP), as well as weather temperature, while qualitative measures were established from the PANAS questionnaire, and included age, height, medication, alcohol status, weight, and smoking status. This study recruited 45 (n) healthy adults, including 18 female and 27 male participants. The modes of commute were bus (n = 8), driving (n = 6), cycling (n = 7), train (n = 9), tube (n = 13), and both bus and train (n = 2). The participants wore non-invasive wearable biosensor technology to measure EEG and blood pressure during their morning commute for 5 days in a row. A correlation analysis was applied to find the significant features associated with stress, as measured by a reduction in positive ratings in the PANAS. This study created a prediction model using random forest, support vector machine, naive Bayes, and K-nearest neighbor. The research results show that blood pressure and EEG beta waves were significantly increased, and the positive PANAS rating decreased from 34.73 to 28.60. The experiments revealed that measured systolic blood pressure was higher post commute than before the commute. For EEG waves, the model shows that the EEG beta low power exceeded alpha low power after the commute. Having a fusion of several modified decision trees within the random forest helped increase the performance of the developed model remarkably. Significant promising results were achieved using random forest with an accuracy of 91%, while K-nearest neighbor, support vector machine, and naive Bayes performed with an accuracy of 80%, 80%, and 73%, respectively.

## 1. Introduction

Commuting to work or school is often a daily activity that comprises a significant amount of time and effort. Full-time workers in England spend an average of one hour per day commuting, and one in seven workers spends at least two hours commuting. People’s commuting ways are influenced by their personal characteristics and life circumstances [1]. The commuter’s daily commute may affect them in both objective and subjective ways while traveling, after traveling, and over time. Commuting can be time-consuming, costly, and unpleasant, and it can have an impact on mood both during and after the journey. The effect that commuting will have on your health will probably depend on how long you commute, what kind of transportation you use, and the weather you encounter [1]. Recent research analyzed commute stress among commuters who used various modes of transportation and discovered that those who walked or cycled to work experienced the least stress and those who drove experienced the most stress [2].

The human brain reacts to various moods and emotions. The brain is like a control center, and it usually uses electrical impulses to control how the body works. Numerous environmental factors, including exercise, anxiety, sleep, and stress, affect the heart rate. According to Peter et al., stress in any form in humans can cause a change in heart rate and BP [3]. It has been shown that cutting down on free time and sleep makes people less productive because it throws off their daily schedules. Commuters reported higher levels of mental stress, more health issues, the majority of which were psychosomatic in nature, and more sick days taken from work [4].

According to Milner, Kavanagh, Badland, and LaMontagne, there is a threshold relationship that governs how much time is spent commuting, for more than 6 h per week is linked to declining subjective mental health outcomes. Job control and job security are moderating factors in this relationship [5]. The study discovered that when commuting time exceeded 6 h per week, people who were considered to demonstrate low levels of control in their professional function received negative ratings on a mental health questionnaire. Longer commutes resulted in higher stress levels for commuters, depending on how much their travel was affected, for example, by unreliable public transportation and traffic, according to O’Regan’s findings [6]. More evidence has shown that the degree of control a commuter has over their travel affects how much of an impact longer commutes have on stress [7]. It has been proposed that traffic congestion, rather than necessarily the amount of time spent traveling, is the mediator of stress during commutes due to their strong correlation, especially in urban regions [8].

Research used body mass index, hip circumference, waist circumference, and waist–hip ratio to predict high blood pressure [9]. This investigation was carried out using a machine learning technique called classification trees. Machine learning techniques are widely employed in many fields for a variety of purposes [10]. Similarly, research was conducted to detect stress in working people using ECG and galvanic skin response (GSR). According to a study, stress is a mental health issue that can induce depression, loss of clarity at work, bad working relationships, socio-financial issues, and in some extreme situations, suicide. EEG signals were recorded using sensor equipment, they were pre-processed and used to investigate the stress level using the machine learning techniques SVM and KNN. Datasets included both sensor data and information on the stress of the workplace, such as email interruptions and deadline pressure [11]. A new method for evaluating stressed and depressed people’s psychophysiology has emerged in recent years because of the measurement and objectification of stress [12]. Researchers collected data from a diverse range of users in around 100 nations using a well-known meditation app, including heart rate variability (HRV) and heart rate (HR), besides the self-assessment of stress [13]. Those data were recorded using a mobile camera as a standalone or as a voluntary pair before and after the assessments.

Our daily lives are significantly impacted by stress because it is linked to the various tasks we complete daily. Our body responds to outside events in several ways, both directly and indirectly. Monitoring bio signals such as pulse rate, blood pressure (BP), and others can teach us about what is happening to our body. Any abnormalities from the previously mentioned factors lead to illness. An individual experiences stress when engaging in any kind of physical or mental effort. Based on bio signals and questionnaire-based surveys, mental stress can be predicted. In a study, stress was detected with machine learning and deep learning using multimodal physiological data [14].

In the present context, there are several technologies to monitor stress levels. These technologies are based on different bio signals: heart rate, heart rate variability, electroencephalograms, blood pressure, skin conductance, cortisol, and pupil diameter. Out of these bio signals, most of the research is based on EEG signals to monitor stress [15]. The emotional and physical health of long-distance commuters is negatively impacted. Additionally, it may cause depression and increased anxiety [16]. Stress can also occur when people have high expectations for someone, but that person is unable to meet those expectations.

Currently, the only people who can tell if someone is stressed or not are medical and physiological professionals. Using a questionnaire is one of the conventional methods for determining stress [17]. This approach solely relies on the participants’ reactions; whether they tremble indicates whether they are under stress. Stress can be found automatically, which can improve social well-being and reduce the risk of health problems. It is important to make a smart model that can predict stress levels using the bio signals. This study uses machine learning to predict how a commute will impact a person’s health, so they can use active or alternative commuting methods to avoid health risks such as high blood pressure or worry.

We aimed to investigate the subjective and objective experiences of the commute. This study examines the social and neurophysiological impacts of real-time commuting. It accomplishes this by utilizing machine learning to analyze brain waves and bio signals. Today’s society has made commuting a necessity and unavoidable part of every day. In this research, we will create an innovative model that investigates the health impacts of commuting using bio signals and an intelligent approach.

## 2. Related Literature Review

Stress is what happens to our bodies when changes in the environment upset the balance of our bodies, minds, or emotions. Positive and negative stress are referred to as eustress and distress, respectively [18]. Stress over a prolonged period can be the cause of several mental health issues. Stress can cause a variety of health problems, including cardiovascular disease [15]. The recognition of mental stress in its earliest stages is very necessary for effective clinical treatment [18]. Most people commonly feel stress in their everyday lives as a typical physiological reaction to their environment [16]. People typically experience it when they feel intimidated or challenged. People get the impression that it is challenging to balance and adjust to both internal and external situations. Several wearable technologies, such as Olive, Spire, and Breath Acoustics, can detect the degree of tension in the user [19].

Standard surveys or questionnaires are the foundation of most stress detection studies. Such a technique requires a significant amount of time and resources, as a specialized individual must evaluate the stress in each one [16]. Recently, bio-signal-based stress analysis has been developed, which can help by saving time and effort [18]. Bio signals such as HRV, galvanic skin reaction, electrocardiogram, electromyography, blood pressure, and finger and skin temperatures are used for real-time stress identification [20].

Similarly, a study was conducted to identify stress while driving using medical data, including heart rate, ECG, breathing, and EMG under different circumstances. Biodata such as respiration, GSR from the hand and foot, heart rate, and EMG were analyzed in this study utilizing a machine-learning-based technique. The information was then divided into time periods of 100, 200, and 300 s for varying degrees of stress. The segmented data was then used to create statistical characteristics, which were then given to the chosen classifier. Stress levels were labelled based on their intensity as high, medium, and low. SVM exceeded KNN with an accuracy of 98.41% for time intervals of 100 s and 200 s and 99.1% for intervals of 300 s [21]. Cho et al. also investigated how deep neural networks and ECG data might identify stress levels [22]. They used a cross-validation method to evaluate their model.

A questionnaire was used to classify the stress of university students using various machine learning techniques: linear regression, SVM, naïve Bayes, and random forest [23]. In this research, the support vector machine performed well compared to other techniques. Rizwan et al. used ECG signals to detect stress levels [18]. Various SVM methods were applied to alter the feature number and types of kernels. With an accuracy of 98.6%, the gaussian cubic SVM produced a very promising result.

Pascual et al. made a portable device that can measure how stressed a person is [24]. This device uses different bio signals as input parameters, such as body temperature, heart rate, and galvanic skin responses. Those bio signals were classified using an artificial neural network algorithm, which performed very well, with an accuracy of 91.67% [25].

The EEG data were analyzed offline with a LabView-based algorithm to monitor the degree of stress that was being experienced [26]. In that model, EEG was recorded using a one-channel EEG headset, and the recorded EEG signals were obtained from the headset using the EEGID mobile application. According to their findings, when people are under stress, the EEG beta band will be greater than the alpha band. In a similar study, Jaun and Ioannis assessed emotion, mood, personality, and social situation using EEG data and the PANAS form (individual vs. group setting) [27]. This was completed with an SVM technique in which the characteristics of each EEG band’s differential entropy and fractal dimension were found. According to their study, these characteristics were connected to participants’ emotional reactions. They addressed the two situations that served as the labels for their dataset. According to their findings, employing SVM with a radial basis function for their arousal scenario resulted in an accuracy of 68%, while using SVM with a linear kernel function for their emotional valence scenario delivered an accuracy of 61%.

Using an end-to-end learning approach for the time and spatial dimensions related to EEG data, a novel EEG classification network was developed in the research to enhance classification performance [22]. The techniques described an architecture with 77.9%, 89.91%, and 88.31% accuracy in motor imagery and emotion classification, respectively. Similarly, a group of researchers developed a non-invasive brain–computer interface to identify and categorize human mental states using EEG data [28]. In that study, the continuous decoding of EEG data was used to classify the flying-related mental states of pilots. Various simulated flying scenarios were used to collect the EEG data from seven pilots.

In past studies that tried to build models to predict stress levels, blood pressure and HRV were the two most important factors. Compared to bio signals, physiological characteristics are given less thought. Aside from artificial neural networks, the most popular machine learning applications are support vector machines and K’s closest neighbor. In this research, we are predicting that systolic BP will be higher when the EEG beta low power exceeds alpha low power after the commute.

## 3. Data Collection and Research Hypothesis

**Figure 1:**
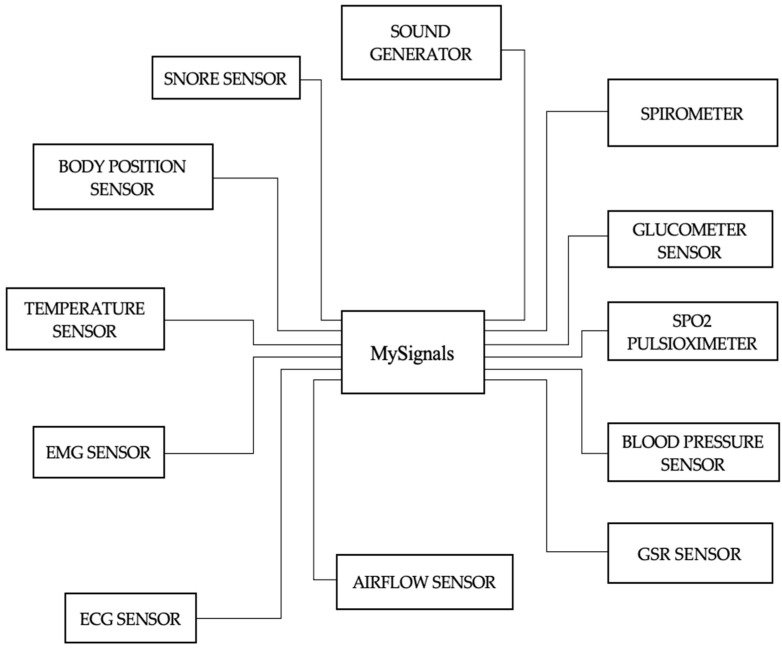
MySignals with sensors.

The employed data in this study were collected from 45 individuals who were in generally good health and who travelled to London each day for their jobs for five days in a row. Each participant completed an informed consent form after being cleared medically to take part in this study. According to the demographics in Table 1, participants in this data-gathering procedure came from various parts of London, worked at various places, and travelled to work using a variety of modes, where frequency refers to the number per category. In this study, multimodal data were used, including responses from a questionnaire as well as numerous human bio signals including blood pressure, heart rate, and EEG signals (PANAS). Bio signals were collected using non-invasive wearable biosensor technology. As shown in Figure 1, the MySignals device was utilized to capture the user’s blood pressure as well as their heart rate both before and after the journey. Blood pressure was collected from the upper arms before and after the commute. We can monitor more than 20 bio signals with this device, including heart rate, BP, ECG, oxygen levels in the blood, pulse, and breath rate. Similarly, EEG signals were captured from many points on the head while the subject was travelling from their house to their workplace. Additionally, the individuals’ heart rates and blood pressures were measured and recorded. Through the collection and analysis of bio signal datasets that were gathered throughout the journey from home to work, we were able to examine the effects that commuting within Greater London had on the participants’ mood and stress levels. These datasets were collected during the commute from home to work. The recording of bio signals was an extremely risk-free process that posed no danger to the participants in the study.

The major steps in the data collection were as follows:

- While travelling to work, participants had to wear an EEG headset, and a sensor arm and ear clip.
- For five working days, bio signals were recorded when they commuted to work.
- They were instructed to initialize the devices and fill out an online health questionnaire as a part of the pre-experiment process.
- The wearable devices captured the bio signals when the participant was travelling to and from work.
- When the participant arrived at their place of work, they took three to four minutes to complete an online survey that documented their experience during their commute.
- Please keep in mind that all the information or data that was recorded was made anonymous and kept secret.

Similarly, the participants in this study consisted of people who lived in London, were employed, and often commuted there for five consecutive working days. To calculate the necessary sample size for this study, we utilized a simple random sampling approach. This method gave each person an equal chance of being selected for this research. Daniel WW utilized the technique to determine the sample size for this research presented in [29]. Using the simple random sampling method, the sample size for our research was found to be 42. Additionally, similar research was conducted with a much smaller number of participants compared to our research. The research was conducted to detect levels using machine learning and deep learning using multimodal physiological data from only 15 subjects [3]. Similarly, another study, in which machine-learning-based signal processing using physiological signals was used for stress detection, was conducted successfully using data from only 17 drivers [21].

**Figure 2:**
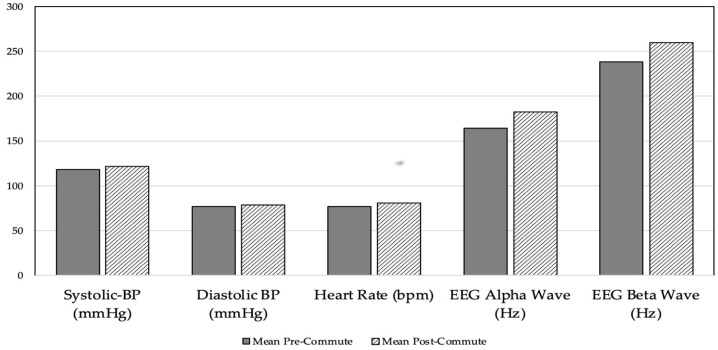
Comparison of pre- and post-commute blood pressure and heart rate.

Every participant’s blood pressure and heart rate were taken for five days in a row using the MySignals device. Blood pressure is made up of two numbers: the first is systolic BP and the second is diastolic BP. The pressure that is present while the heart contracts is known as systolic pressure, while the pressure that is present when the heart relaxes in between beats is known as diastolic pressure. Blood pressure is measured in millimeters of mercury (mmHg). The ideal blood pressure ranges from 90/60 mmHg to 120/80 mmHg. The top number is systolic, and the bottom number is diastolic blood pressure. Similarly, heart rate and EEG power spectrum are expressed as beats per minute (BPM) and hertz (Hz), respectively. The value of the EEG band in Figure 2 is plotted with a sampling rate of every 10 s. These data were collected from the participants pre commute and post commute. The observed patterns of bio signals show that heart rate and blood pressure are generally higher post commute than pre commute, as shown in Figure 2.

**Figure 3:**
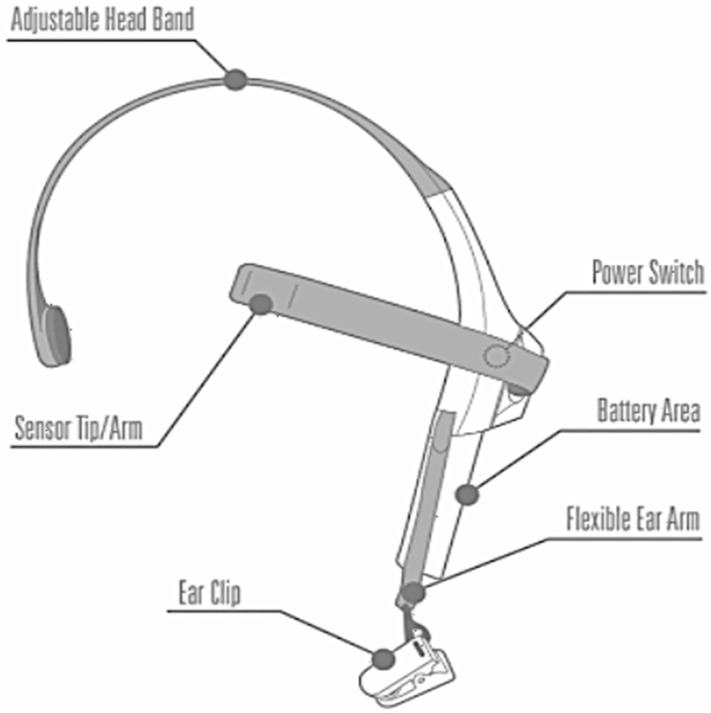
EEG mobile headset.

Similarly, EEG data were acquired using a mobile EEG headset throughout the journey to work, as illustrated in Figure 3. This headset measures and securely produces EEG power spectrums. A headset, an ear chip, and a sensor arm were all included in this set of equipment. The reference and ground electrodes of the headset were attached to the ear clip, as shown in Figure 3, and the EEG electrode was attached to the sensor arm, which was placed on the forehead above the eye (FP1 position). One AAA battery was all that was needed for its eight hours of operation [22].

The data collection stages also involved the collection of other subjective factors and parameters from every participant including age, height, gender, weight, medication intake, smoking and alcohol status, location, weather temperature (degree Celsius), and medical health. Blood pressure can vary or rise for a variety of causes, such as when people consume excessive alcohol or medication with a high sodium/protein content, or when they have low levels of calcium, potassium, or magnesium [30]. A PANAS questionnaire form was used to collect the responses from the participants before and after the commute [31]. The PANAS questionnaire was created in 1988 based on research work from the University of Minnesota and Southern Methodist University. This questionnaire comprises a scale of words that, according to a person’s surroundings, indicate their feelings and emotions [32]. The PANAS scale ranges from 1 to 5, as shown in Table 2 and Table 3.

In this research, two datasets were formed using the main data. The first dataset comprised objective parameters (BP and EEG), while the second dataset comprised both objective parameters (BP and EEG) and additional parameters including age, gender, height, medication intake, weight, and smoking and alcohol status. We had to pre-process the data before applying the chosen machine learning algorithms. Pre-processing of data was conducted based on the hypothesis. The data analysis was conducted to visualize and identify any null values or missing data. There were 225 rows, which comprised the data collected from 45 participants over 5 days (45 × 5). Similarly, there were 11 columns, which represented the different objective parameters. To employ the chosen algorithms, the data for each algorithm was pre-processed to evaluate the study hypothesis. Based on the characteristics, each dataset was classified as “X” or “Y” (training data or target data). Datasets were split into training and test sets at a ratio of 80:20. Samples were labelled as zero (class 0) and one as positive (class 1). The samples were labelled as positive (class 1) if the EEG beta low power was higher than the EEG alpha power and the systolic BP had increased after the commute; otherwise, they were labelled as zero (class 0).

To employ the different machine learning algorithms, the pre-processed data for each method was used to evaluate the following research hypotheses: After the commute, if the EEG beta low power is greater than the alpha low power, systolic blood pressure will be higher. In this research, the hypothesis was tested to detect stress levels after commuting. EEG and blood pressure were acquired from the participants during the commute. The EEG signal may be utilized to detect and track stress levels in humans [26]. EEG data consists of five distinct bands: delta, theta, alpha, and beta. In this research, we only used alpha and beta bands, as the alpha band is associated with when we are relaxed, while the beta band is active when we are actively thinking, alert, or stressed. Similarly, systolic pressure was selected from the collected BP, as it occurs when the heart contracts to pump blood out. Additionally, it is regarded as a stronger predictor of stress compared to diastolic pressure [33,34].

## 4. Implementation

A machine-learning-based approach was designed to implement and execute the data analysis. A comprehensive experiment was performed to come up with the best machine learning approach’s structure that was suitable for the data in question. The machine learning techniques chosen in this research are SVM, naive Bayes, KNN, random forest, and MLP neural network. Measures for evaluating the performance of the model included the area under the curve (AUC) and the receiver–operator characteristic (ROC) curve [35]. ROC curves illustrate a classification model’s performance at each categorization level. Equations (1) and (2) show how these two variables, false positive rate (FPR) and true positive rate (TPR), are represented:

(1) $$TPR=\frac{TP}{TP+FN}$$

(2) $$FPR=\frac{FP}{FP+TN}$$

The effectiveness of all the chosen machine learning approaches was summarized using a confusion matrix [36]. The confusion matrix’s main job was to show with count values and by class how many predictions were right and how many were wrong. The confusion matrix shows the effectiveness or precision of a classification model.

In the same way, cross-validation was used to avoid making decisions that were biased because the model was tested with all the empirical data [37]. It is the process of folding data into K folds, where K stands for the maximum number of folds that may be achieved. The test set for each partitioned dataset was used, while the training set was used for the other partitions. The divided data was then used as a test set at least once for each part. Performance was assessed using the mean value. Compared to the conventional method of splitting data into training and test sets, it showed less variability.

### 4.1. Performance Metrics for Classification

#### 4.1.1. Confusion Matrix

For machine learning classification issues, this performance assessment metric is most frequently utilized. This matrix measures M by M, where M is the total number of classes and labels [36]. A confusion matrix presents a table layout of the different outcomes of the prediction and the results of a classification problem and helps visualize its outcomes. It plots a table of all the predicted and actual values of a classifier. Calculating predictive accuracy is made possible by comparing actual and expected results. True positives are described as situations where the predicted and actual class values are equal (1, 1). False positives are described as situations where the predicted class value is 1 while the actual class value is 0. When the predicted result is 0 but the actual value is 1, this is referred to as a false negative. A real negative situation is one in which the actual and predicted numbers are the same (0, 0).

#### 4.1.2. Classification Accuracy

The model’s classification accuracy is the percentage of correct predictions it makes [38]. TP, TN, FP, and FN stand for true positive, true negative, false positive, and false negative, respectively. According to Equation (3), it is the proportion of all accurate predictions (true negatives with true positives) to all guesses.

(3) $$Accuracy=\frac{\left(TP+TN\right)}{\left(TP+TN+FP+FN\right)}$$

#### 4.1.3. Precision

This is the proportion of all positive instances that are correct out of all positive instances that were expected. In many instances, we cannot rely on accurate categorization, such as when we have an unbalanced dataset in which one class is more prevalent than the others. In such a scenario, a model’s accuracy will be high if it predicts just the most common class for all outputs. This sort of categorization is ineffective since the model does not acquire any knowledge. As illustrated in Equation (4), precision is computed to determine the fraction of valid positive predictions [39].

(4) $$Precision=\frac{TP}{TP+FP}$$

#### 4.1.4. Recall

The rate at which true positives are classified is often referred to as the true positive rate. According to Equation (5), support measures each label’s actual replies [40].

(5) $$Recall=\frac{TP}{TP+FN}$$

#### 4.1.5. F1 Score

The F1 score includes both recall and accuracy [40]. It may be determined using the harmonic mean of both measurements. It is the calculation of the classifier’s precision and robustness in label classification. It ranges from 0 to 1 and corresponds to low and high scores, as demonstrated by Equation (6).

(6) $$F1\;Score=2\times \frac{Precision\times Recal}{Precision+Recall}$$

#### 4.1.6. The Area under ROC Curve

It is the region that the ROC curve covers [41]. The classifier with the largest area under the curve performs the best. Another statistic that illustrates the trade-off between recall and precision is the area under the accuracy-recall curve. Its values range from 0 to 1, with 1 being the highest attainable score.

## 5. Results and Discussion

### 5.1. Approach 1: Using Only EEG, and BP Data

#### 5.1.1. Support Vector Machine

**Figure 4:**
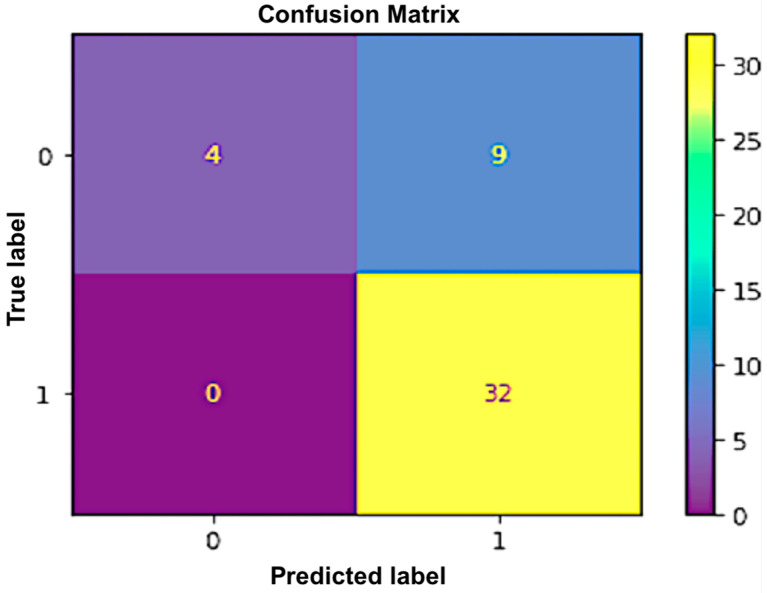
Confusion matrix for SVM.

The support vector machine is a well-known tool for machine learning because it can deal with both regression and classification problems. In the SVM classifier, we used a medium-gaussian SVM as the kernel function. The kernel scale used was 5.2, as it gave the highest accuracy. One-vs-one was used as a multi-class method. We did compare using the different values, When the kernel value was increased or decreased from 5.2, the accuracy of the model decreased. After applying the SVM to classify whether systolic BP will be high or low when the EEG beta low power exceeds alpha low power after the commute, we obtained an accuracy of 80%. Applying that algorithm, 32 of the 41 predicted values were correctly classified, while nine were misclassified. Similarly, four out of four objects in the second class were accurately categorized. The total accuracy obtained from this algorithm is shown in the following Figure 4.

**Figure 5:**
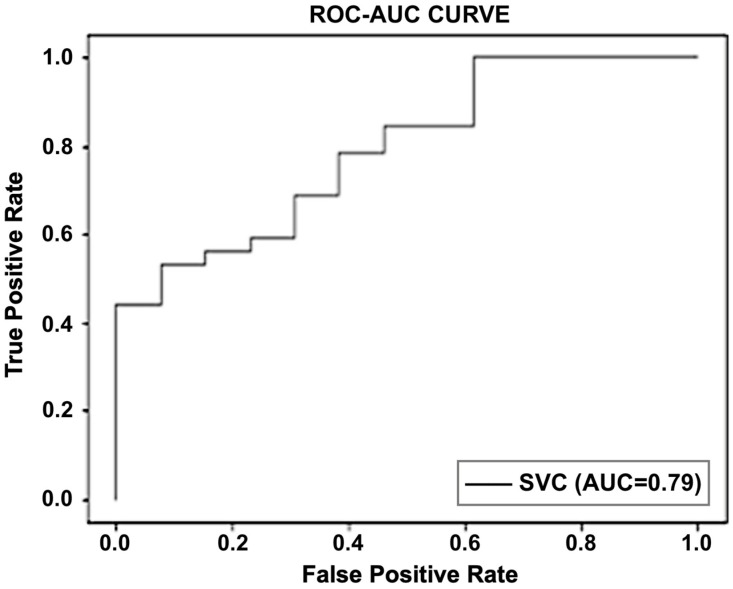
Graphical representation of the performance of SVM using a ROC curve.

Similarly, ROC curves were also created to assess the effectiveness of algorithms. In contrast to most other measures, they offer a graphical picture of a classifier’s performance. The performance of the SVM utilizing the ROC curve is shown in Figure 5.

#### 5.1.2. K-Nearest Neighbor

**Figure 6:**
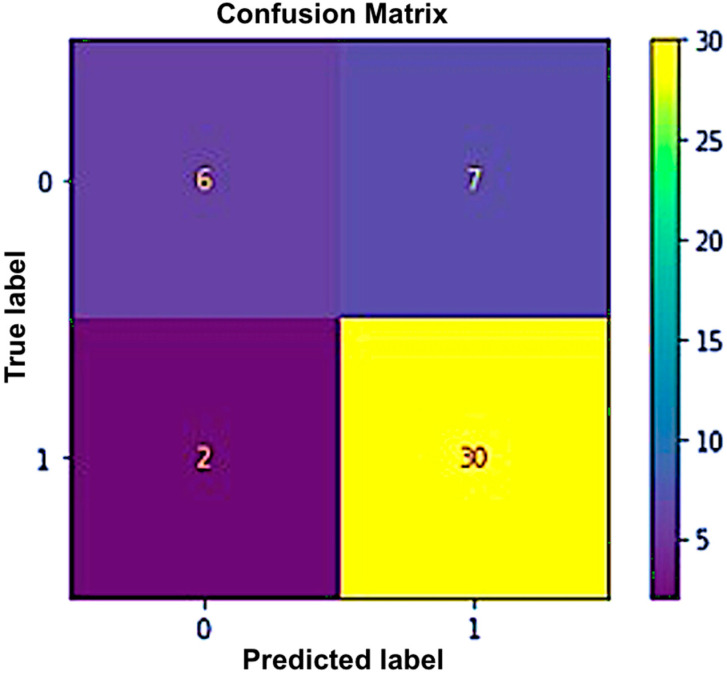
Confusion matrix for the K-nearest neighbor.

**Figure 7:**
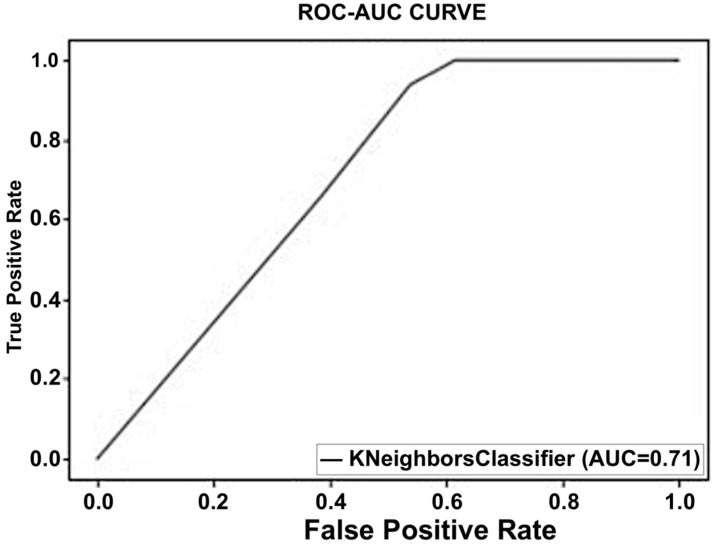
Performance of the K-nearest neighbor using the ROC curve.

A supervised learning technique called KNN is commonly applied to regression and classification problems. A given input is categorized using this non-parametric technique based on its neighbors. This model determines the separation between the supplied data point and each training input. The test input is subsequently allocated to the class of the K-nearest neighbors. In classification and regression issues, KNN—which classifies an input based on its nearest neighbor—is frequently utilized. The distance between the provided data point and all training inputs is computed by this model. The test input is subsequently assigned to the class of its K-nearest neighbor. For this classifier, we used the weighted KNN with a value of K = 10. In that model, the distance metric was Euclidean, and distance weight was squared inverse. We tried the model with different values of K; we obtained the highest accuracy for K = 10. Using this algorithm, we obtained an accuracy of 80% for the first dataset. The confusion matrix as shown in Figure 6 reveals that the classifier correctly predicted 30 out of the 37 right values, while seven were misclassified, whereas for the second class, six out of eight values were properly categorized. Similarly, Figure 7 depicts a graphical depiction of this classifier’s performance using the ROC curve.

#### 5.1.3. Random Forest

**Figure 8:**
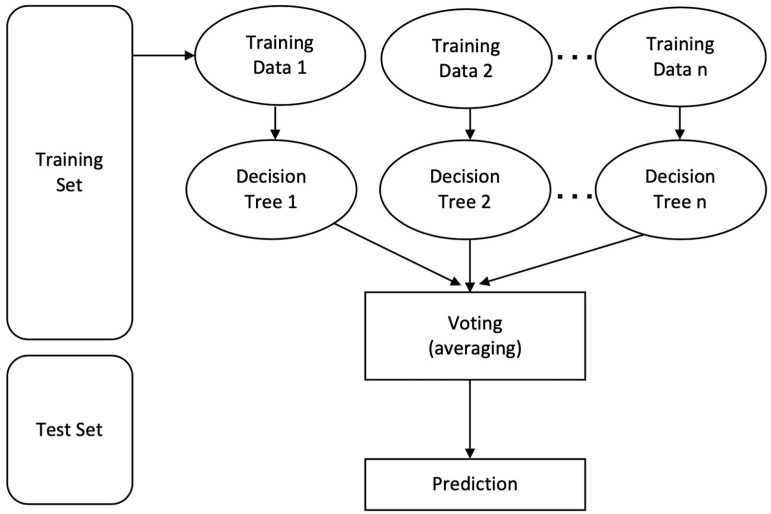
The random forest algorithm.

Each decision tree in the collection predicts the specified class. The outcome of the model is determined by considering the outputs of all decision trees. This algorithm combines many models to improve performance. An accuracy of 91% was the greatest result achieved by the random forest. Figure 8 illustrates the operation of the random forest method.

The following stages provide a detailed explanation of the random forest algorithm:

- Select samples at random from a data or training set.
- This algorithm will produce a decision tree for each training set.
- Voting will use an average of the choice tree.
- Choose as the final prediction outcome the one that has received the most votes.

#### 5.1.4. Naive Bayes

The classifier naive Bayes is a Bayes-theorem-based supervised learning method. The underlying premise is that the presence of one character in one class has no bearing on the existence of another feature in a different class. The probability of occurrence is computed using all possibilities, regardless of their interdependence. This classifier is optimal when the dataset contains several characteristics and has a high dimensionality. We have used the Gaussian naïve Bayes algorithm in this research as we have continuous data and data with a normal distribution, for example, age, height, and weight. It is a basic and quick classification algorithm capable of making rapid predictions. Using this method, we obtained an accuracy of 77%.

#### 5.1.5. Multi-Layer Perceptron Neural Network

MLP is an additive neural network for feedforward. The data for processing are provided to the neural network’s input layer. The output layer is responsible for categorization and prediction. Given that there is a probability range of 0 to 1, the sigmoid activation function was utilized in this neural network.

A neural network’s input layer receives data to analyze, while the output layer predicts and categorizes the data. The initial hypermeter to adjust the neural network is the number of neurons in each hidden layer. Three layers make up the MLP classifier, one of which is hidden. In the MLP classifier, the sigmoid activation function is used in the ANN. The sigmoid activation function was used in this neural network as the probability exists between 0 and 1. It is especially used for models where there is a designed model that must predict the probability. There were 28 epochs, and the best performance was received at epoch 22. Using this technique, we achieved an accuracy (Acc) of 73%. Similarly, implementation was carried out with and without cross-validation. Then, the results obtained were compared, as shown in Table 4.

### 5.2. Approach 2: Using EEG, BP, and Personalized Parameters

Similarly, we used all the chosen machine learning algorithms on the second dataset to create a model that predicts the physiological impact of commuting. The second dataset contains both primary parameters (heart rate, blood pressure, and EEG) and subjective parameters (age, weight, height, gender, any medicine consumed, smoking, alcohol intake, location, weather temperature (degrees), and medical health).

#### 5.2.1. Random Forest

**Figure 9:**
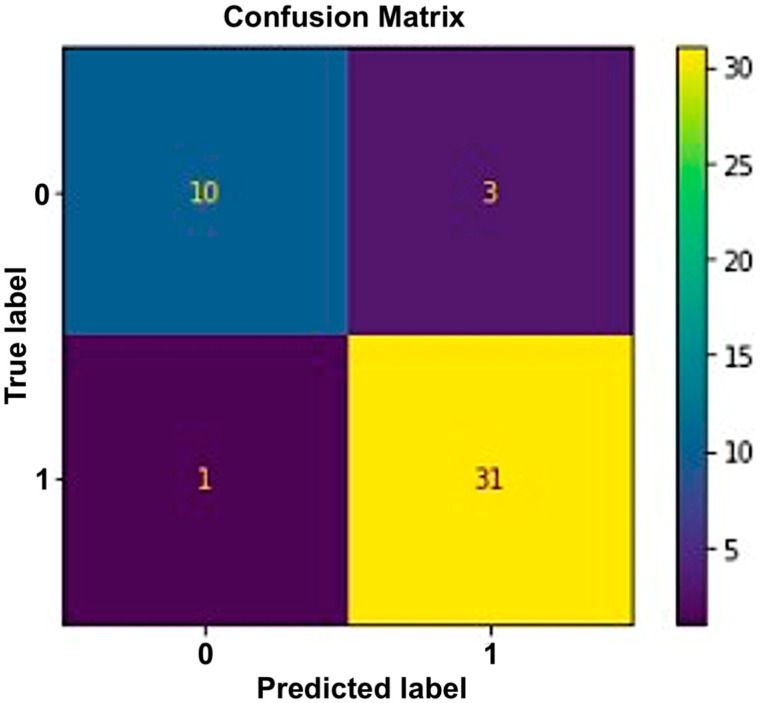
Confusion matrix for random forest.

Gini was utilized as a splitting criterion in this strategy. The nodes were expanded until all leaves were pure or had fewer than the minimum samples with a 100-tree estimator number and a 0-maximum depth. As pruning is a suitable approach to reducing overfitting, we used the cross-validation method to check for overfitting. We compared this with different combinations of the above parameters and obtained the best result with Gini as the splitting criterion, none as the maximum depth, and 100 as the number of trees. Random forest performed very well with an accuracy of 91%, as shown in Figure 9.

**Figure 10:**
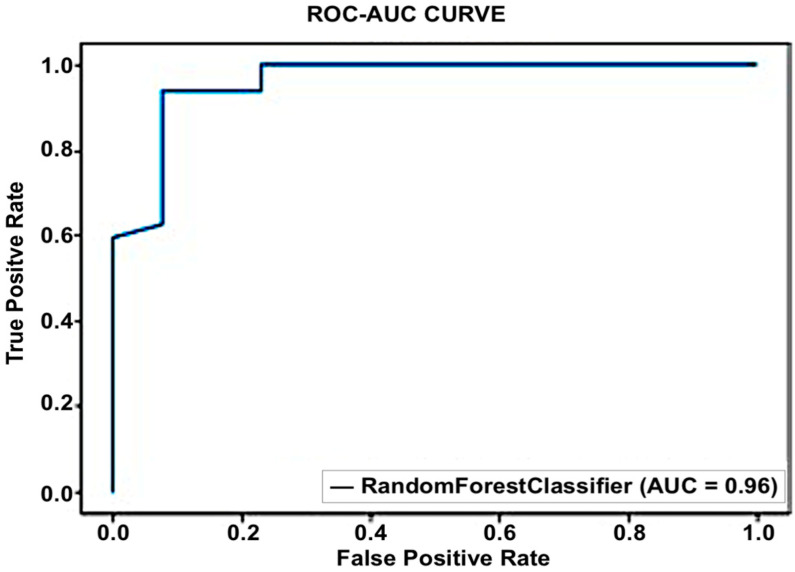
Performance of random forest using ROC Curve.

Similar to this, ROC curves were employed to evaluate the effectiveness of algorithms. In contrast to most other measures, they offer a graphical picture of a classifier’s performance. Figure 10 displays the random forest’s performance.

#### 5.2.2. Naive Bayes

**Figure 11:**
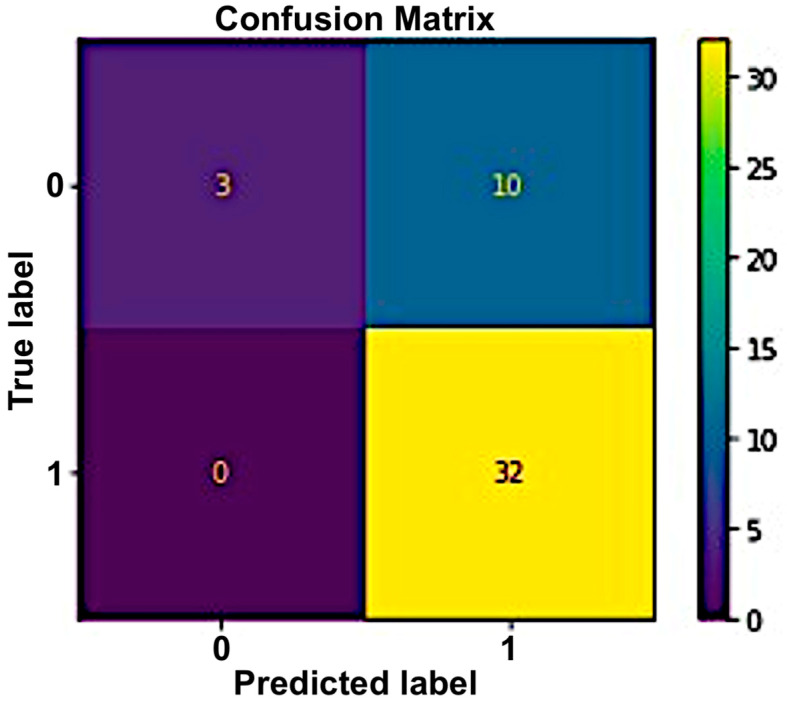
Performance of naive Bayes using the confusion matrix.

**Figure 12:**
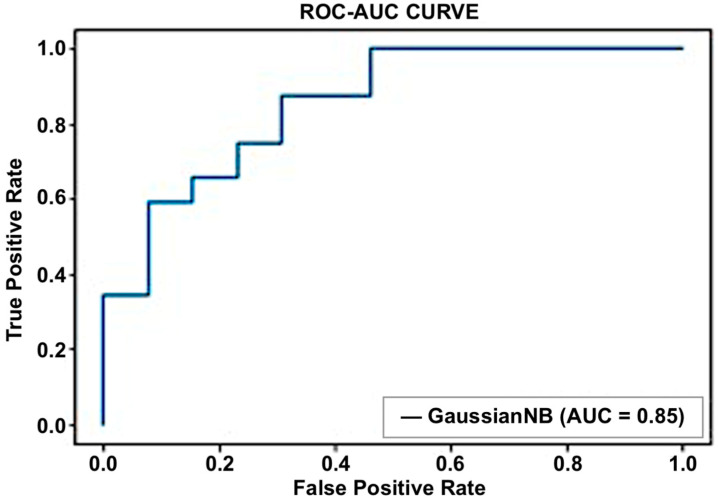
Performance of naive Bayes using the receiver–operator characteristics (ROC) curve.

In this method, Gaussian naive Bayes performed well compared to other naive Bayes classifiers. Gaussian was used as a distribution name for numeric predictors. An accuracy of 78% was achieved using this classifier. Figure 11 and Figure 12 show the confusion matrix and ROC curve calculated to display the performance of this classifier.

Similarly, when the support vector machine was applied to the second dataset, it performed well with an accuracy of 80%. There was no change in the performance when applied to either the first or second dataset. K-nearest neighbor and multi-layer neural network gave an accuracy of 78% and 76% when applied to the second dataset which also comprised personalized parameters.

Additionally, there were two classes for the accuracy, recall, and F1-score, with class 0 being a negative class and class 1 being a positive class. The values were computed to check the accuracy of the prediction of true and false values in each class. For example, if the value of precision class 0 was 1 that signified that 100% of the data were classified correctly. We can use accuracy when we are interested in correctly predicting both 0 and 1 and our dataset is balanced enough. We utilized recall to identify as many actual 1 as was feasible, while precision was employed to ensure that the forecast of 1 was as accurate as possible. The F1 score is also a weighted average of recall and accuracy. The output of several methods, when used on the second dataset, is shown in Table 5.

## 6. Analysis and Critical Review

### 6.1. Approach 1: Using Only the Main Objective Parameters (EEG, and BP)

**Figure 13:**
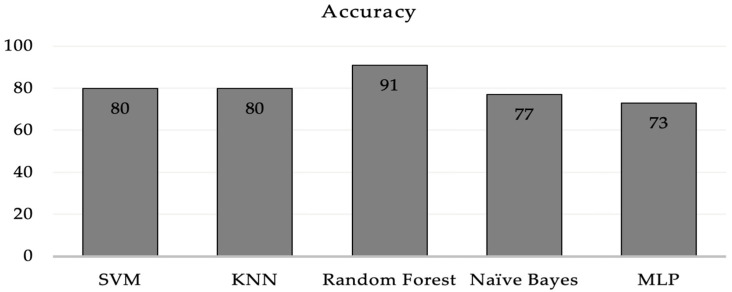
Comparison of the performance of different machine learning algorithms.

Using this method, we used several artificial intelligence (AI) methods on the first set of data, which had EEG and BP as the main parameters. SVM and KNN classifiers came in second and third, respectively, with an 80% and 91% classification accuracy, outperforming all other methods. Figure 13 shows that compared to the other options, the MLP classifier works the worst.

**Figure 14:**
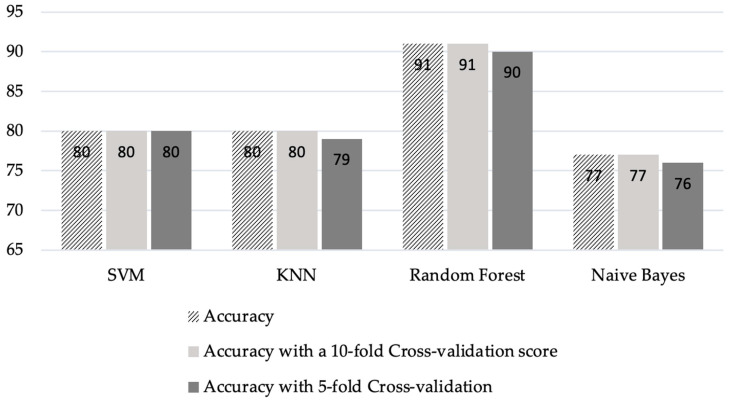
Classification with cross-validation.

By utilizing cross-validation, the degree of precision changes little. When the K-fold cross-validation approach was completed with K = 5 and K = 10, the accuracy of the SVM classifier remained unaffected; however, the MLP classifier was significantly affected compared to other algorithms. As shown in Figure 14, the random forest provided the best degree of accuracy, which was 91%.

### 6.2. Approach 2: Using EEG, BP, and Personalized Parameters

We applied the second dataset to this method. The second dataset included the key parameters (EEG and BP) as well as age, height, smoking, weight, alcohol intake, heart rate, and morning weather temperature. For the second dataset, random forest obtained the highest accuracy score of 91%.

### 6.3. PANAS Results

In this research, a PANAS questionnaire was used to collect responses from the participants before and after the commute. This questionnaire comprised a scale of words that according to a person’s surroundings, indicated their feelings and emotions. The PANAS scale ranges from 1 to 5, as shown in Table 2 and Table 3 above. All the participants filled out the form before and after the journey. Using this form, we calculated the positive and negative affect scores before and after the commute. A positive affect score is calculated by adding the scores on lines 1, 3, 5, 9, 10, 12, 14, 16, 17, and 19 from Table 2. The scores range from 10 to 50. A higher value indicates higher levels of positive affect. Similarly, a negative affect score is calculated by adding the items 2, 4, 6, 7, 8, 11, 13, 15, 18, and 20 from Table 3. The negative affect score also ranges from 10 to 50. Bigger values indicate higher levels of negative affect. Once the positive and negative affect scores were calculated, we averaged the pre-positive affect, pre-negative affect, post-positive affect, and post-negative affect for all the participants. Table 6 presents the average positive and negative affect before and after commuting.

In Table 6, the positive affect score before the commute is higher compared to the positive affect score after the commute, which indicates that the participants’ feelings and emotions were more positive before the commute. Similarly, the negative affect score increased after the commute, indicating that the participants were less stressed before the commute. It also indicates that participants were more positive or interested in going to work before the commute.

## 7. Conclusions

This study developed several machine-learning-based approaches deploying multimodal data to create an intelligent model capable of predicting the commute impact on human health. This research acquired responses to a questionnaire (PANAS) to illustrate the effect of commuting on self-reported evaluations. The employment of a machine-learning-based approach has led to the conclusion that systolic blood pressure was greater post commute. It was independent of the length of the journey and whether it was a short or long period of time. After the commute, beta low power in the EEG was found to be greater than alpha low power. Alpha power is associated with a relaxed but awakened state, which is observed when we are awake but relaxed and not processing a great deal of information, whereas beta power is associated with a state of mental and intellectual activity and outwardly focused concentration, reflecting a state of alertness.

An accuracy of 91% was achieved for the first dataset, which contained the objective parameters (EEG and BP). While a 91.1% accuracy rate was attained for the second set of data, which included objective parameters and personalized parameters. The random forest algorithm showed the best performance in both datasets. The results obtained from the selected classifiers supported the research hypothesis. Similarly, using PANAS, we also found that positive affect was higher before the commute. This means that the participants were more positive and interested in going to work before the commute. Negative affect was higher after the commute, which means that participants were less interested or more stressed. The objective results from the machine-learning-based method supported the subjective results obtained using PANAS.

The goal of this study was to find out how commuting in a busy city affects a person’s body and try to predict what those effects are based on machine learning approaches. It also helps recording the experience of commuters with a special focus on the use of emerging computing technologies. This research will help make a living lab for multimodal research experiments in areas such as body sensors, ubiquitous computing, and wireless telehealth. Currently, the only people who can tell if someone is stressed or not are medical and physiological professionals. Using a questionnaire is one of the conventional methods for determining stress. This approach solely relies on the participants’ reactions: whether they tremble indicates they are under stress or not. Stress can be found automatically, which can improve social well-being and reduce the risk of health problems. It is important to make an intelligent model that uses people’s body data to automatically figure out how stressed they are and prevent hazards to their health such as high blood pressure. The objective bio signals (heart rate and BP) were found to be higher post commute than pre commute, regardless of the commute duration. Mood and stress are favorably linked with bio signals. Based on the machine learning technique, we were able to determine the participants’ stress levels after commuting.

Our future work will focus on designing a smart model to find out how commuting affects productivity at work.

## Figures and Tables

**Table 1:** The demographic information of the study population.

Category	Sub-Category	Number (N)
Sex	Male	27
	Female	18
Age	Less than 25	7
	Between 25–45	30
	45+	8
Location	North London	11
	Southeast London	21
	East London	11
	Southwest London	2
Mode of Commute	Bus	8
	Driving	6
	Cycling	7
	Train	9
	Tube	13
	Bus and Train	2

**Table 2:** Positive and Negative Affect Schedule (PANAS) scale.

1	2	3	4	5
Very Slightly or Not at All	A Little	Moderately	Quite a Bit	Extremely

**Table 3:** Positive and Negative Affect Schedule (PANAS) scorecard.

1. Interested 2. Distressed 3. Excited 4. Upset 5. Strong 6. Guilty 7. Scared 8. Hostile 9. Enthusiastic 10. Proud	11. Irritable 12. Alert 13. Ashamed 14. Inspired 15. Nervous 16. Determined 17. Attentive 18. Jittery 19. Active 20. Afraid

**Table 4:** Comparison of different algorithms with and without cross-validations.

Approach	Accuracy	Acc with 10-Fold Cross Validation Score	Acc with 5-Fold Cross-Validation
SVM	80	80	80
KNN	80	80	79
Random Forest	91	91	90
Naïve Bayes	77	77	76
MLP	73	71	64

**Table 5:** Comparison of the performance of different algorithms using different performance metrics.

Technique	Accuracy	Cross-Validation Score	Precision Class 0	Precision Class 1	Recall Class 0	Recall Class 1	F1 Class 0	F1 Class 1
SVM	80	0.71	1.0	0.78	0.31	1.0	0.47	0.88
KNN	78	0.73	0.67	0.81	0.46	0.91	0.55	0.85
Random Forest	91	0.75	0.91	0.91	0.77	0.97	0.83	0.94
Naïve Bayes	78	0.73	1.0	0.76	0.23	1.0	0.38	0.86
MLP	76	0.64	0.60	0.80	0.46	0.88	0.52	0.84

**Table 6:** Comparison of positive and negative affect scores before and after the commute.

Avg Pre-Positive Affect	Avg Post-Positive Affect	Avg Pre-Negative Affect	Avg Post-Negative Affect
34.73	28.60	11.16	19.05

## Data Availability

Restrictions apply to the availability of these data. The data are not publicly available due to these restrictions.
